# Attenuation of acute lung inflammation induced by cigarette smoke in CXCR3 knockout mice

**DOI:** 10.1186/1465-9921-9-82

**Published:** 2008-12-16

**Authors:** Li Nie, Ruolan Xiang, Weixun Zhou, Bao Lu, Deyun Cheng, Jinming Gao

**Affiliations:** 1 Department of Respiratory Disease, Peking Union Medical College Hospital, Chinese Academy of Medical Sciences & Peking Union Medical College, Beijing 100730, PR China; 2 Department of Respiratory Disease, West China Hospital, Sichuan University, Chengdu 610041, Sichuan Province, PR China; 3 Department of Pathophysiology, Peking University Health Sciences Center, Beijing 100088, PR China; 4 Department of Pathology, Peking Union Medical College Hospital, Chinese Academy of Medical Sciences & Peking Union Medical College, Beijing 100730, PR China; 5 Ina Sue Perlmutter Laboratory, Division of Pulmonary, Children's Hospital, Harvard Medical School, Boston, MA 02115, USA

## Abstract

**Background:**

CD8+ T cells may participate in cigarette smoke (CS) induced-lung inflammation in mice. CXCL10/IP-10 (IFNγ-inducible protein 10) and CXCL9/Mig (monokine induced by IFN-γ) are up-regulated in CS-induced lung injury and may attract T-cell recruitment to the lung. These chemokines together with CXCL11/ITAC (IFN-inducible T-cell alpha chemoattractant) are ligands for the chemokine receptor CXCR3 which is preferentially expressed chiefly in activated CD8+ T cells. The purpose of this investigation was to study the contribution of CXCR3 to acute lung inflammation induced by CS using CXCR3 knockout (KO) mice.

**Methods:**

Mice (n = 8 per group) were placed in a closed plastic box connected to a smoke generator and were exposed whole body to the tobacco smoke of five cigarettes four times a day for three days. Lung pathological changes, expression of inflammatory mediators in bronchoalveolar lavage (BAL) fluid and lungs at mRNA and protein levels, and lung infiltration of CD8+ T cells were compared between CXCR3-/- mice and wild type (WT) mice.

**Results:**

Compared with the WT littermates, CXCR3 KO mice showed less CS-induced lung inflammation as evidenced by less infiltration of inflammatory cells in airways and lung tissue, particularly fewer CD8+ T cells, lower levels of IFNγ and CXCR3 ligands (particularly CXCL10).

**Conclusion:**

Our findings show that CXCR3 is important in promoting CD8+ T cell recruitment and in initiating IFNγ and CXCL10 release following CS exposure. CXCR3 may represent a promising therapeutic target for acute lung inflammation induced by CS.

## Background

Exposure to cigarette smoke (CS) is a major risk factor for the pathogenesis of chronic obstructive pulmonary disease (COPD) [1]. CS initiates the infiltration of innate and adaptive inflammatory cells into the airways and the lung parenchyma and further destroys the alveolar structure [2-6]. The role of inflammation in the development of COPD is supported by the finding of excess numbers of CD8+ T cells in lung tissues from patients with COPD and an inverse relationship to the lung function [7,8]. CD8+ T cells in epithelium and submucosa expressing CXCR3 were increased in numbers in smokers with COPD as compared with nonsmokers. Excessive CD8+ T cells in the lungs of COPD also produce large amounts of IFNγ and IFNγ-induced CXC chemokines, such as CXCL10/interferon-inducible protein-10 (IP-10). CXCL10, a CXCR3 ligand, was abundantly expressed in bronchiolar epithelial cells and airway smooth muscle cells [9,10]. CXCL9/Mig, CXCL10/IP-10, CXCL11/ITAC are chemokines that attract activated T cells through binding to their receptor, CXCR3 [11]. Collectively, these findings suggest that CXCR3/CXCL10 interaction may play a pivotal role in the pathogenesis and progression of COPD through T cell recruitment in airways and lung parenchyma.

A key initiating event in COPD is the recruitment of inflammatory cells into the lung in response to CS exposure [1], which is regulated by a variety of chemokines [9-11]. CXCL9/Mig, CXCL10/IP-10 and CXCL11/ITAC, ligands for CXCR3, and CCL5/RANTES, ligand for CCR5, were shown to be elevated in sputum from COPD patients compared with nonsmokers [12]. Chemokine receptors have been implicated in the pathogenesis of lung inflammation in rodent models exposed to CS. For example, CXCR2 has been demonstrated to be involved in acute pulmonary inflammation induced by CS [13]. In CCR5 gene ablated mice, lung tissue inflammation and apoptosis induced by IFNγ and CS were also significantly decreased [14,15]. Recent reports demonstrated that CCR6, together with its ligand CXCL20/MIP-3α, was involved in CS-induced lung inflammation and that the interaction between CCR6 and CCL20/MIP-3α could also mediate accumulation of dendritic cells (DCs) in the lungs of COPD patients [16,17].

In terms of CXCR3, particularly expressed on activated Th1/Tc1 cells [9,10,16-18], we hypothesized that CXCR3 gene deficiency would terminate or at least attenuate CS-induced pulmonary inflammation and tissue damage. To address this hypothesis, we used CXCR3 KO mice and their WT littermates to investigate the contribution of CXCR3 in CS-induced lung injury process.

## Methods

### Mice and cigarette smoke exposure

CXCR3 gene deficient mouse line has been established by gene targeting as described elsewhere [19]. CXCR3 KO mice and WT littermate mice (Experimental Animal Research Center, Beijing, China) with C57BL/6 background (backcross > 14 generations), were maintained in a pathogen-free mouse facility at Peking Union Medical College. Clean food and water ad libitum were given. Ten to 12 week old mice (~20–22 grams of weight) were used in the experiments.

A commercially-available filter cigarette was used (White Shark brand, Tobacco Company, China), and according to manufacturer's specification, each cigarette contained 1 mg of nicotine and 13 mg of tar. CS exposure was performed according to previously-described methods [13,15,16]. Briefly, mice were placed in a closed plastic box connected to smoke generator. The mice (n = 8 per group) were exposed whole body to the tobacco smoke of five cigarettes four times a day with 30-minute smoke free interval for three consecutive days. Control mice were received filtered air according to the same procedure. Animals were killed on the fourth day after CS exposure by pentobarbital overdose.

All experiments were performed according to international and institutional guidelines for animal care, and approved by Peking Union Medical College Hospital Committee on Animal Care and Use.

### Histological analysis of lung tissue

The mice were sacrificed and the lungs were removed, inflated to 25 cmH_2_O with 10% formalin and fixed overnight, embedded in paraffin, and sectioned at 5 μM. Hematoxylin & eosin staining was performed at the Department of Pathology, Peking University Health Sciences Center. The pathological analysis was independently performed in each mouse in a blind manner by two pathologists.

### Bronchoalveolar lavage (BAL)

Mice were sacrificed, and the trachea was cannulated by using a 20-gauge catheter. BAL was performed twice with 0.8 ml of ice-cold PBS (pH 7.4) each. 1.5 ml of total injected volume was recovered in >95% of mice. The BAL fluid was spun at 1500 rpm for 5 min at 4°C, and supernatant was collected for the measurement of cytokines and total protein. The pelleted cells were harvested, and red cells were lysed, then the pelleted cells were washed and resuspended in cold PBS. Total cells were enumerated by counting on a hemocytometer. For differential cell counting, cells were spun onto glass slides, air-dried, fixed in ethanol, and stained with Diff-Quick reagents (Baxter Scientific, Miami, FL). The number of macrophages, neutrophils and lymphocytes in 400 cells was counted based on morphology.

### Lung homogenates

Animals were euthanized and perfused with 3 ml of cold saline via the heart. The left lobes were removed and homogenized in 1 ml of PBS containing complete protease inhibitor cocktail (Sigma, St. Loius, MO). Then, the samples were centrifuged for 10 min at 3000 rpm. Supernatants were filtered through a 0.45 μm filter and kept in -70°C until used.

### Preparation of lung single-cell suspensions

The lungs were excised, minced and enzymatically digested for 30 min in 15 ml of digestion buffer (RPMI, 10% FBS, 1% penicillin/streptomycin, 1 mg/ml collagenase (Sigma) and 30 μg/ml DNase (Sigma, St Louis, MO). The undigested fragment was further dispersed by repeated passage through a Nytex filter. The total cells were pelleted, and any contaminating red cells were lysed by ice-cold hypotonic RBC solution. After spinning, the pellet was resuspended in 10 ml of completed medium (RPMI 1640, 10% FBS, 1% penicillin/streptomycin). An equal volume of 40% Percoll (Sigma, St Louis, MO) was added, and the cells were spinned at 3000 rpm for 30 min at room temperature. The cell pellets were resuspended in complete medium, and leukocytes were counted on a hemacytometer in the presence of 0.4% trypan blue. Cells were >90% viable by trypan blue exclusion. Cytospins of recovered cells were prepared for differential staining as described above.

### Labeling cells from BAL fluid and single lung cell suspensions from lung tissue

50 ul of 2 × 10^7^/ml of cells from BALF and collagenase digested lung cells was used. 10 μl of blocking buffer (1 μl blocking antibody Fc in 9 ml PBS/2%BSA) was added to the cells for 15 min on ice to block nonspecific binding. After washing once, cells were incubated with 50 μl of FITC-conjugated anti-CD4 Ab and PE-conjugated anti-CD8 Ab or control mouse IgG2b (BD PharMingen, San Diego, CA) for 1 hr on ice. Cells were washed twice by PBS and fixed in PBS containing 2% formalin. Cells were subjected to flow cytometer on a FACScan (Coulter).

### Determination of protein content in BAL fluid

Total protein content in BAL fluid was measured using the BCA Protein Assay Kit (Pierce, Rockford, IL) according to manufacturer's instructions.

### ELISA analysis of IFNγ and CXCL10

The concentrations of IFNγ, and CXCL10 (the limit of detection were 12.5 pg/ml and 2.2 pg/ml, respectively) in BAL fluid and lung homogenates were determined by ELISA kits (R& D systems) according to manufacturer's recommendations.

### RNA extraction and semi-quantitative RT-PCR analysis

Total RNA was extracted from the lung using TRIzol reagent (Invitrogen) according to manufacturer's instructions, and treated with RNase-free DNase. RNA was reverse-transcribed and cDNA was subjected to PCR for analyzing the expression of IFNγ, CXCL9, CXCL10, CXCL11, granzyme A, granzyme B, perforin, and β-actin. The primers and conditions for PCR are detailed in Table 1.

**Table 1:** RT-PCR primers, conditions and products

**RT-PCR Genes**	S/AS	Primer sequence (5' to 3')	Tm(°C)	Product (bp)
**CXCL9**	S	CTTGGGCATCATCTTCCT G	55	352
	AS	TGAACGACGACGACTTTGG		
**CXCL10**	S	GTCATTTTCTGCCTCATCC	55	273
	AS	GAGCCCTTTTAGACCTTTT		
**CXCL11**	S	CTGCTCAAGGCTTCCTTATGTT	55	166
	AS	CCTTTGTCGTTTATGAGCCTTC		
**IFNγ**	S	CATCTTGGCTTTGCAGCTCTT	55	363
	AS	CTGGACCTGTGGGTTGTTGA		
**Granzyme-A**	S	GAAACCAGGAACCAGATGC	55	390
	AS	GTGACAGGGATGGAGTGAA		
**Granzyme-B**	S	CCCTCTGCCTTCTTCCTC	55	344
	AS	CTGGGTCTTCTCCTGTTCTT		
**Perforin**	S	ATGGCACGCACTTTATCAC	55	413
	AS	CTTCGGGTTCTGTTCTTCC		
**β-actin**	S	CTTCCTTAATGTCACGCACGATTTC	55	541
	AS	GTGGGGCGGCCCAGGCACCA		

S, sense; AS, antisense

### Statistical analysis

Data are expressed as means ± SEM. As appropriate, comparisons between two groups were carried out using ANOVA and Student's *t *test (two-tailed) using GraphPad PRISM software (Version 4.0 for windows; GraphGrad, San Diego, CA). A value of *P *< 0.05 was considered significant.

## Results

### Inflammatory cells are reduced in CXCR3 KO mice exposed to CS

**Figure 1:**
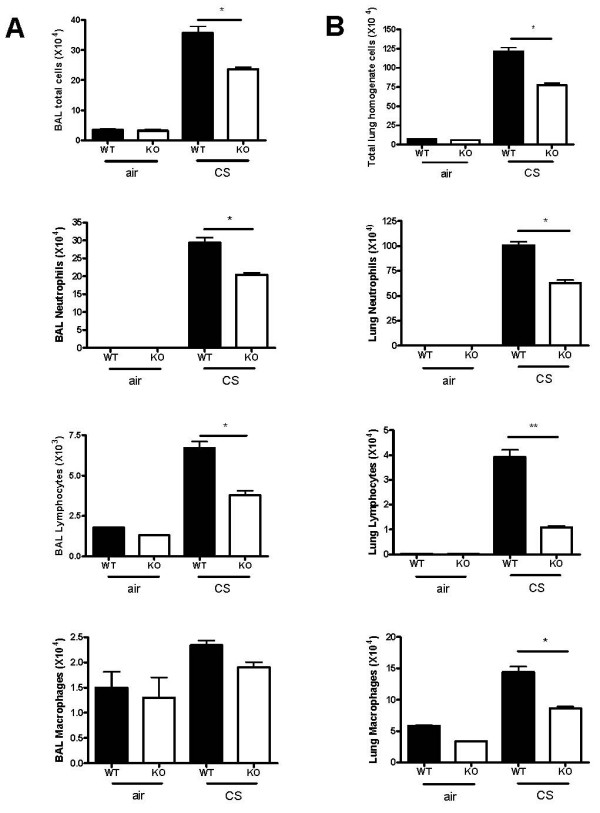
Effect of CXCR3 gene deficiency on infiltration of inflammatory cells in mice after 3 days of CS exposure. Panels A and B, Total inflammatory cells and differential populations recovered from BAL fluid and lung homogenates. Results are expressed as means ± SEM, n = 5–8 animals per group, *, *p *< 0.05.

To determine whether CXCR3 deficiency affects the CS-induced infiltration of inflammatory cells into airways and parenchyma, we estimated the cell subpopulations in BAL fluid and lung tissue following CS exposure. There was significantly less infiltration of inflammatory cells into airways in CXCR3 KO mice compared with WT mice, except for macrophages (figure 1A). Consistently, the numbers of total inflammatory cells and differential subpopulations harvested from lung parenchyma were significantly decreased in CXCR3 KO mice group than in WT mice (figure 1B).

**Figure 2:**
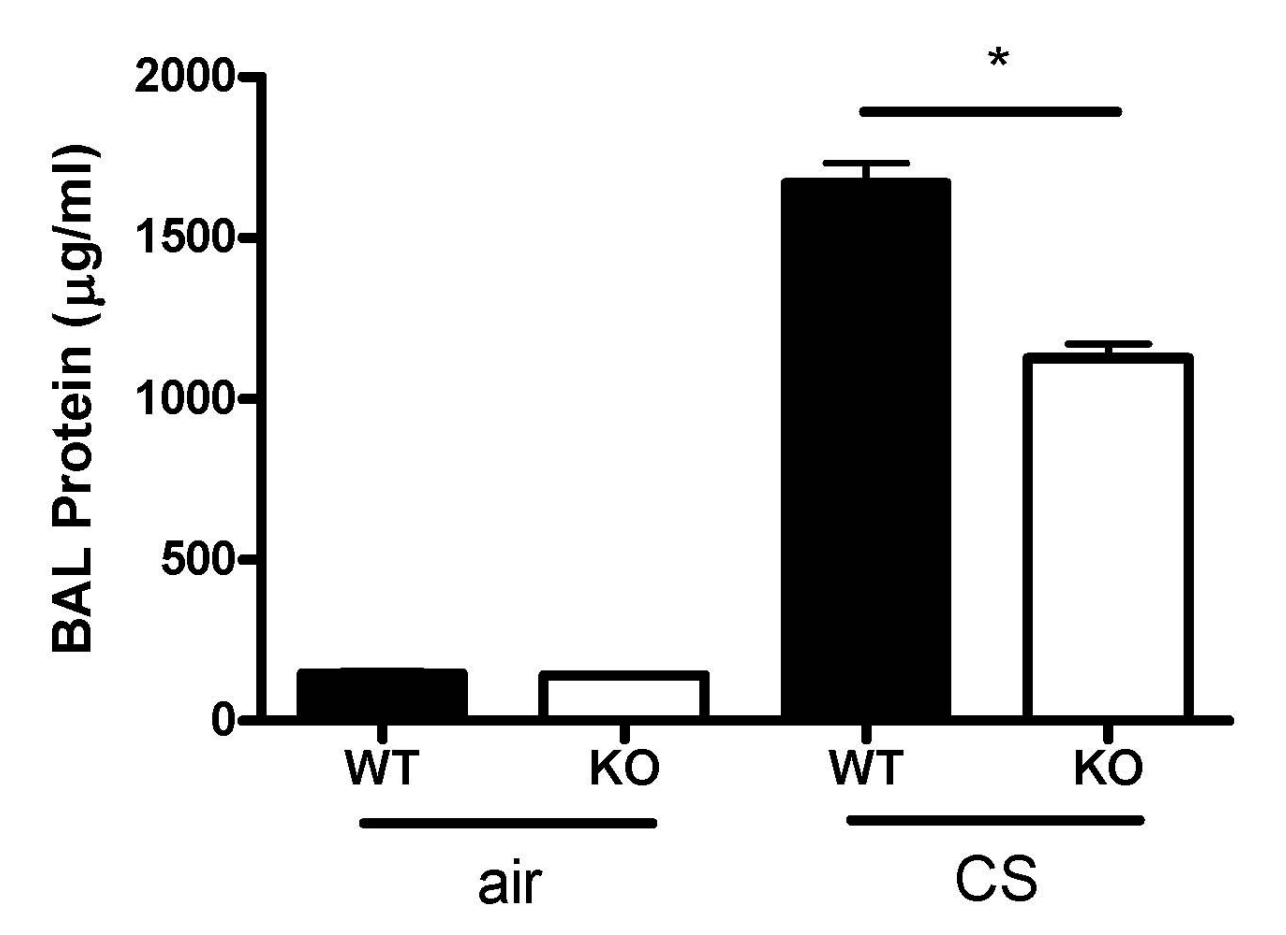
Effect of of CXCR3 gene deficiency on protein leakage from circulation to airways after 3 days of CS exposure. Results are expressed as means ± SEM, n = 5–8 animals per group, *, *p *< 0.05.

Protein leakage was greater in WT mice than in CXCR3 KO mice, indicating that there was more fluid accumulation in alveolar spaces through the damaged alveolar and endothelial cells in WT mice (figure 2).

**Figure 3:**
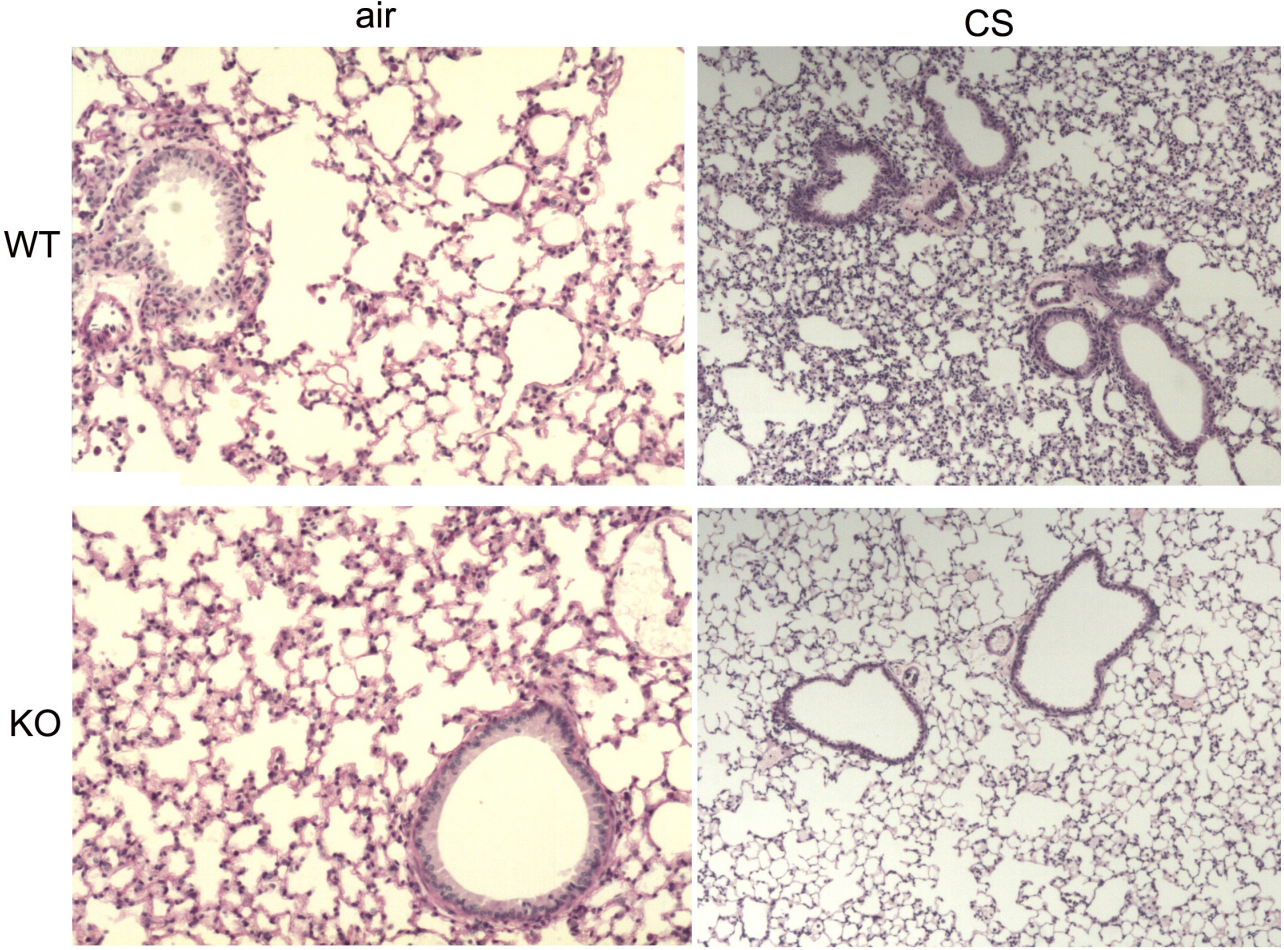
Morphometry of the lungs in CXCR3 KO and WT mice after 3 days of air or CS exposure. Representative photomicrographs of hematoxylin- & eosin-stained lung tissues.

Compared with CXCR3 KO mice, there was a greater aggregation of leukocytes, and distortion of alveolar architecture in WT mice (Fig 3).

### CD8+T cells in airways and lungs in CXCR3 KO mice exposed to CS

**Figure 4:**
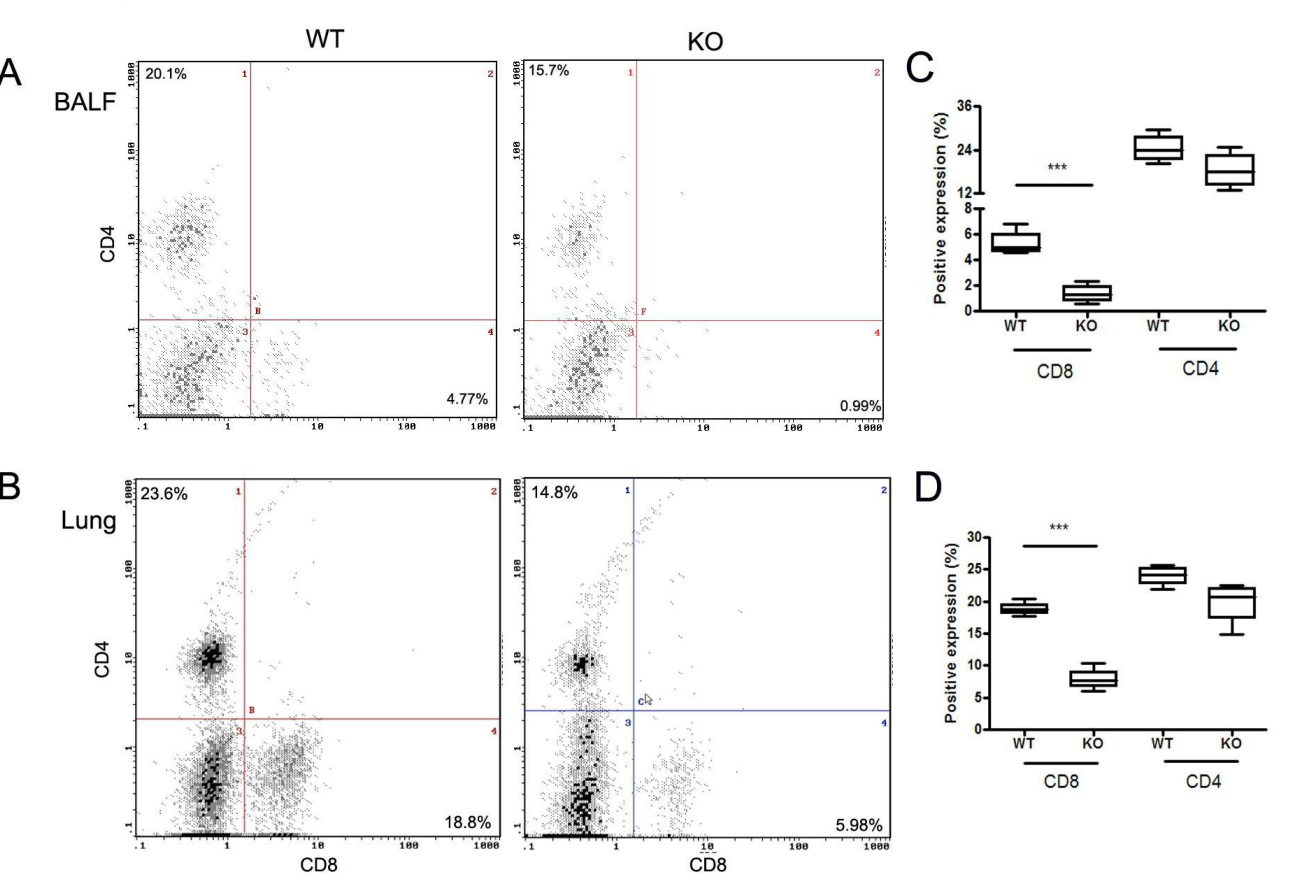
Effect of CXCR3 deficiency on CD8+ T cells and CD4+ T cell infiltration into airways and lungs in CXCR3 KO and WT mice after CS exposure. Panels A and B, representative histograms showing expression of CD4+ T cells and CD8+ T cells in BALF and Lung. Panels C and D, pooled data showing the percentage of CD4+ T cells and CD8+ T cells in BALF and lung. Results are expressed as means ± SEM, n = 4 separate experiments, ***, *p *< 0.001. The data presented are from one representative of four independent experiments.

The percentage of CD8+ T cells in both BAL fluid and lung tissue from CXCR3 KO mice was decreased compared to that from WT mice after CS exposure (BALF: 1.4 ± 0.1% vs 5.4 ± 0.4, p < 0.0001; Lung tissue: 7.9 ± 0.9% vs 18.9 ± 0.5%, p < 0.0001) (figure 4A and 4B). The percentage of CD4+ T cells was similar in both BALF and lungs from WT and CXCR3 KO mice (figure 4A and 4B). In the mice exposed to air, CD4+T and CD8+ T cells were undetectable by FACS analysis (data not shown). These data demonstrate that CXCR3 may be responsible for the initiation of CS-induced inflammation through recruitment of CD8+ T cells, as well as CD4+ T cells, into the airways and lung parenchyma.

### Expression of IFNγ and chemokines

**Figure 5:**
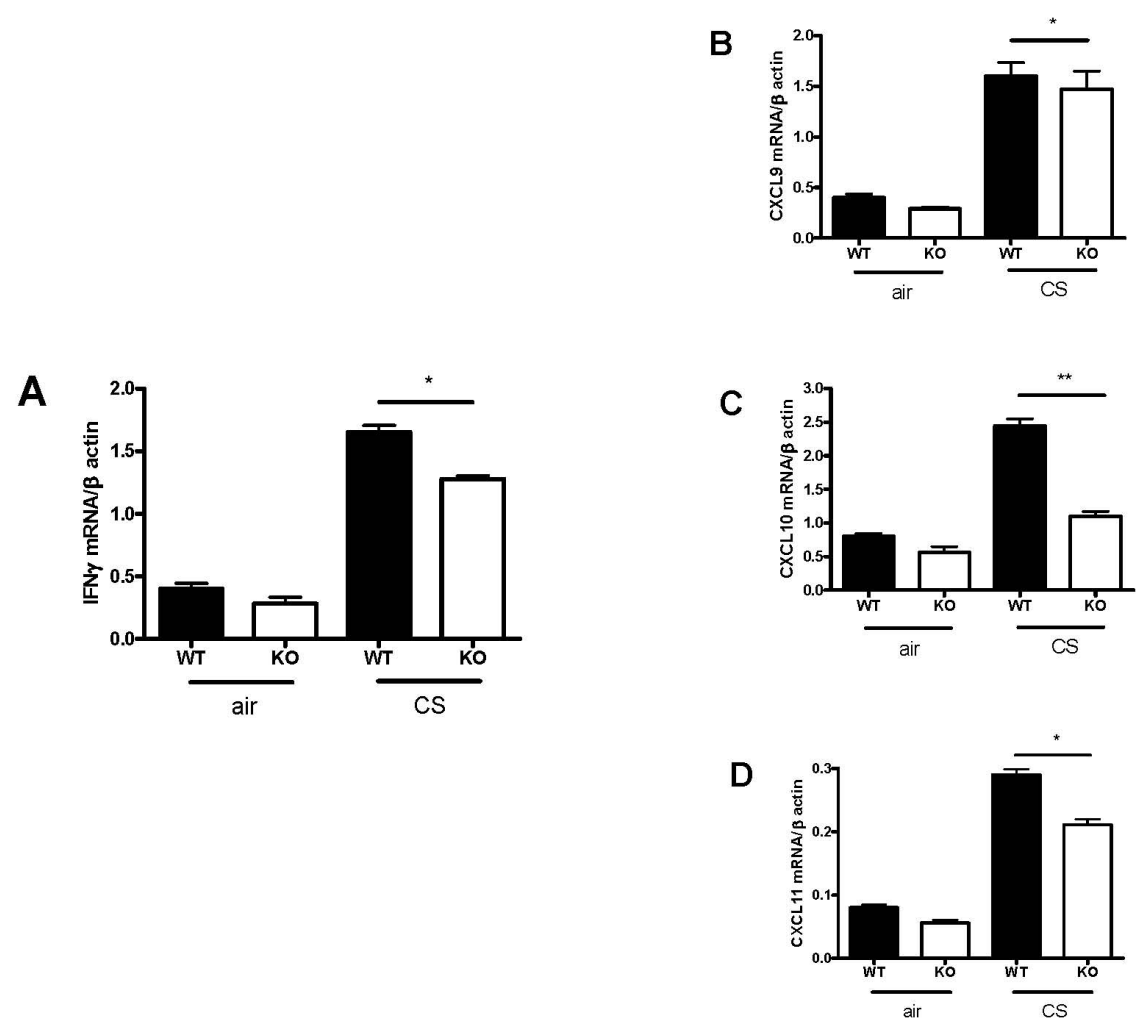
Effect of CXCR3 deficiency on mRNA expression of IFNγ and CXCR3 ligands in lung tissue of CXCR3 KO and WT mice. Panel A, mRNA expression of IFNγ. Panels B-D, mRNA expression of CXCR3 ligands. Results are expressed as means ± SEM, n = 5–8 mice per group, *, *p *< 0.05, **p < 0.01.

The expression of IFNγ mRNA was increased in response to CS in WT mice, but not in CXCR3 KO mice (figure 5A). mRNA expression of CXCL9, CXCL10, and CXCL11 was significantly up-regulated after CS exposure in the lungs from WT mice relative to CXCR3 KO mice (figure 5B–D).

**Figure 6:**
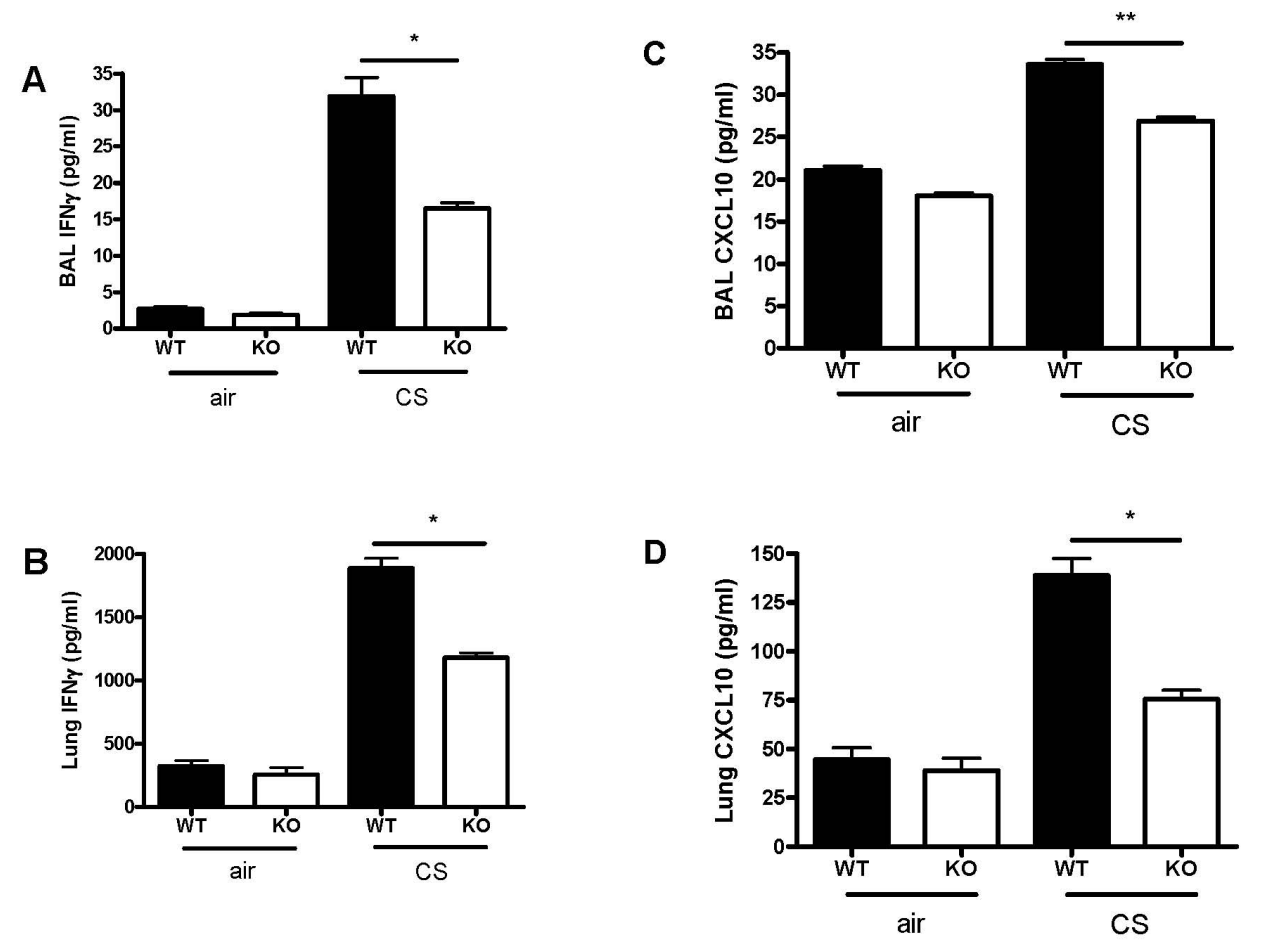
Effect of CXCR3 deficiency on IFNγ and CXCL10 concentration in BAL fluid and homogenates from mice exposed to CS or air for three consecutive days. Panels A-B, IFNγ concentration. Panels C-D, CXCL10 concentration. Results are expressed as means ± SEM, n = 5–8 mice per group, *, *p *< 0.05, **p < 0.01.

The level of IFNγ in BAL fluid and lung homogenates was significantly lower in CXCR3 KO mice than in WT mice (Fig 6A &6B). In addition, CXCL10 concentrations in BAL fluid and lung homogenates were significantly decreased in CXCR3 KO mice compared with WT mice after CS exposure (figure 6C and 6D).

### Expression of granzymes A and B, perforin

**Figure 7:**
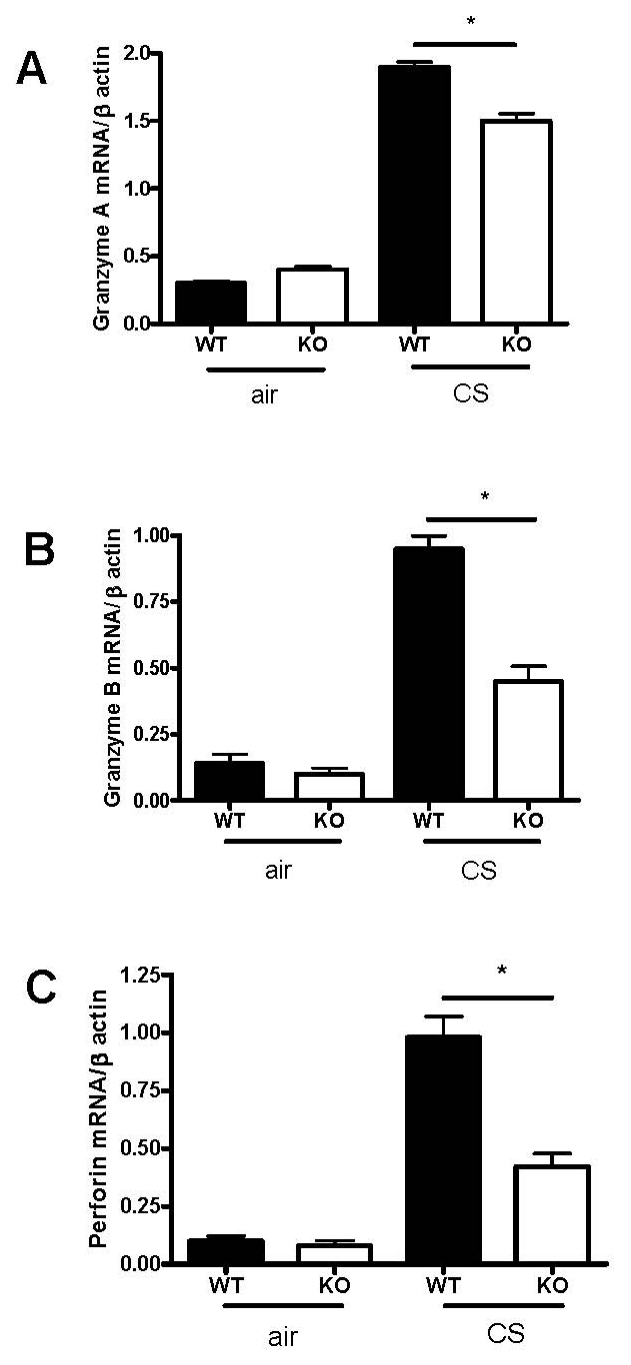
Effect of CXCR3 deficiency on effector molecules of CD8+ T cells in CXCR3 KO and WT mice. Panels A-C, mRNA expression of granzyme A, granzyme B, and perforin in CXCR3 KO and WT mice. Results are expressed as means ± SEM, n = 5–8 mice per group, *, *p *< 0.05.

Upon activation, CD8+ T cells caused cytolysis and apoptosis of alveolar epithelial cells through the release of its effector molecules, including granzymes and perforin [20]. Although mRNA expression for granzymes A, B and perforin was induced in both groups after CS exposure, they were significantly reduced in CXCR3 KO mice as compared with WT mice (figure 7A–C).

## Discussion

In this study, we have demonstrated that deletion of CXCR3 gene in mice significantly prevented the lung inflammation induced by exposure to CS. CXCR3 may be a key factor in CS-induced pulmonary injury by regulating the recruitment of CD8+ T cells as well as other inflammatory cells such as neutrophils and macrophages and by initiating the production of IFNγ, IFNγ-target CXCL10, and the expression of effector molecules from CD8+ T cells.

The local inflammatory response in CS-induced lung injury is associated with infiltration of leukocytes, which is regulated by the members of CXC family [11]. Consistent with previous reports of murine model induced by acute CS exposure [6,13,21], neutrophils represented the majority of cells (~50% of the leukocytes in BAL fluid and lung tissue) in this study. Neutrophilic inflammation is a key factor in the pathogenesis of COPD, and neutrophil infiltration has been shown to be essential for the subsequent recruitment of CD8+ T cells to sites of inflammation [22]. In CS-exposed CXCR3 KO mice, we observed significant reduction in the severity of lung inflammation as evidenced by fewer inflammatory cells in airways and lung tissue and lesser protein leakage into the airway. These observations point to an important role for CXCR3 in the pathogenesis of CS-induced pulmonary inflammation. However, it should be pointed out that CXCR3 KO mice showed partial protection from CS-induced pulmonary inflammation in this study. We have come to realize that CS-induced pulmonary inflammation is not caused by a single chemokine receptor but that multiple chemokine receptors expressed on inflammatory and immune cells are involved [13-16]. Further studies should be done to determine how they interact in a complex network to contribute the pulmonary inflammation caused by CS.

Th1 type cells preferentially express CXCR3 and CCR5, and the infiltrating T cells in COPD express high levels of CXCR3 and CCR5, but not of CCR4 and CCR8 that are preferentially expressed by Th2 cells [2,11,12]. Numerous investigations performed both in vivo and in vitro have consistently found CXCR3 to be associated with Th1/Tc1 responses [2,11,18,23]. In accordance with these findings, we demonstrated that less CD8+ T cells infiltrated into airways and lungs of CXCR3 KO mice. In mice where CD8+ T cells have been deleted, there is resistance to the development of COPD [20]. The explanation for the relative difference in CD8+ T cells between CXCR3 KO and WT mice in this model may in part be due to the downstream effect of CXCR3 activation. Moreover, CD8+ T cells damage the lung interstitium through the release of lytic substances such granzyme A, granzyme B, and perforin [20,24-26]. In support of this notion, we demonstrated that the expression of these enzymes, to some extent, was upregulated in WT mice upon CS exposure. However, the upregulation was inhibited in CS-exposed CXCR3 KO mice. This phenomenon can be attributed to the abrogation of CD8+ T cells in the inflamed lungs from CXCR3 KO mice, which led to the decreased expression of these effector molecules.

The inflammatory response in CS-induced pulmonary damage is characterized by an increased number of Th1 cells, that secrete the Th1-type cytokine, IFNγ [27]. IFNγ expression in BALF and lung homogenates at both mRNA and protein levels was increased after CS exposure either in CXCR3 KO or WT mice, but in CXCR3 KO mice, such increase was blunted. We also demonstrated that CXCR3 ligands were significantly elevated at the transcriptional level required for IFNγ in CS-exposed WT mice. Notably, there was less CXCL10 in both BALF and lung homogenates in CXCR3 KO mice. This can be explained by the negative feedback effect of CXCR3 deletion, in which the reduced accumulation of inflammatory cells in airways and pulmonary parenchyma leads to a diminished release of inflammatory mediators such as IFNγ; more importantly, this leads to inhibition of the activation of airway epithelial cells to produce CXCL10 and decrease in the recruitment of CXCR3 bearing CD8+ T cells [18].

## Conclusion

To our knowledge, this study is the first to specifically focus on the importance of CXCR3 in CS-induced lung inflammation by using CXCR3 KO mice. In conclusion, our study shows that CXCR3 regulates CS-induced lung inflammation via recruitment of CD8+ T cells into the lung to trigger the inflammatory response cascade with over-expression of IFNγ and chemokines that activate CXCR3 ligands, particularly CXCL10. Our findings may provide a therapeutic target for treating CS-induced pulmonary injury.

## Editors' note

Following publication of this article, we have been informed that results from this experiment looking at the effects of cigarette smoke two hours after last exposure, rather than 24 hours as in this article, have been published as: Li Nie, Ruo-lan Xiang, Yong Liu, Wei-xun Zhou, Lei Jiang, Bao Lu, Bao-sen Pang, De-yun Cheng, Jin-ming Gao: *Acta Pharmacologica Sinica* 2008 December; 29 (12): 1432-1439.

## Competing interests

The authors declare that they have no competing interests.

## Authors' contributions

LN and RX performed the whole procedure of the experiments. WZ carried out the pathological analysis. BL and DC helped with designing and drafting the manuscript. JG designed and supervised the experiment, and drafted the manuscript.
